# Relationship between baseline mean platelet volume (MPV) and prognosis of patients with acute mild cerebral infarction undergoing intravenous thrombolysis with Alteplase

**DOI:** 10.12669/pjms.40.3.7493

**Published:** 2024

**Authors:** Yanhao Wang, Xiaoqing Wei, Wei Du, Congcong Yuan

**Affiliations:** 1 Yanhao Wang, Department of Neurology, Baoding No.1 Central Hospital, Baoding 071000, Hebei, China; 2 Xiaoqing Wei, Department of Neurology, Baoding No.1 Central Hospital, Baoding 071000, Hebei, China; 3 Wei Du, Department of Neurology, Baoding No.1 Central Hospital, Baoding 071000, Hebei, China; 4 Congcong Yuan, Department of Neurology, Baoding No.1 Central Hospital, Baoding 071000, Hebei, China

**Keywords:** Mean platelet volume, Thrombolytic therapy, Prognosis, Ischemic stroke

## Abstract

**Objective:**

To investigate the relationship between baseline, mean platelet volume (MPV) and prognosis of patients with acute mild cerebral infarction undergoing intravenous thrombolysis with alteplase.

**Methods:**

A retrospective analysis was conducted of clinical imaging and laboratory data of patients with acute mild cerebral infarction who received intravenous thrombolytic therapy with alteplase in Baoding No.1 Central Hospital between March 2018 and March 2021. According to mRS scores after three months, a total of 140 patients were divided into the good prognosis group(n=115) (mRS score <two) and the poor prognosis group(n=25) (mRS score ≥two). Logistic regression analysis was employed to investigate MPV and other factors that may affect the prognosis. The ROC curve of subjects was used to predict the prognosis of influencing factors. Possible influencing factors of early neurological deterioration (END) were also analyzed.

**Results:**

Logistic regression analysis showed that 24 hour NIHSS score after thrombolysis and baseline MPV were independently correlated with 3-month prognosis. The ROC curve indicated an optimal cutoff value of 9.65 fl for MPV in predicting poor prognosis. The study also found that the increase of baseline MPV had a close bearing on the risk increase of END.

**Conclusion:**

Baseline MPV is an independent risk factor for early neurological deterioration and three months poor prognosis in patients with acute mild cerebral infarction undergoing intravenous thrombolysis.

## INTRODUCTION

Acute Ischemic Stroke (AIS), also known as acute cerebral infarction, is the primary cause of long-term disability in developing countries and death worldwide.^1^ Intravenous thrombolysis with alteplase within four and a half hour of onset is a safe and effective treatment to ameliorate the prognosis.^2^ However, about half of the patients still neither benefit from intravenous thrombolysis nor have a satisfactory prognosis. Therefore, it is of great significance to investigate the related risk factors of poor prognosis of AIS after intravenous thrombolysis for grading the risk of poor prognosis and making clinical treatment decisions for ischemic stroke.^3^

Previous studies have proved that in addition to some clinical factors such as early ischemic signs on images, atrial fibrillation, baseline NIHSS score, some blood test indexes such as homocysteine (Hcy), blood lipid index, platelet index are also important risk factors for poor prognosis of patients with acute cerebral infarction undergoing intravenous thrombolysis.^3^-^5^ Mean Platelet Volume (MPV) is indexes of platelet volume size that are routinely tested in clinical settings and are related to platelet activation.^6^ It has been found in previous studies that the increase in MPV is a risk factor for the development of ischemic stroke and is associated with a poor prognosis of ischemic stroke.^7^,^8^ Several studies in recent years have revealed the critical predictive value of MPV for the prognosis of patients undergoing acute cerebral infarction by intravenous thrombolysis, especially in patients with large atherosclerotic type, but no subgroup analysis of the prognosis of patients with acute mild ischemic stroke by intravenous thrombolysis has been reported.^6^ In this study, the factors associated with the prognosis of intravenous thrombolysis in patients with mild ischemic stroke were investigated, and the correlation between MPV and the prognosis of intravenous thrombolysis in patients with acute mild cerebral infarction.

## METHODS

In this retrospective study, patients with acute mild cerebral infarction who were hospitalized in Baoding No.1 Central Hospital and received intravenous thrombolysis with alteplase between March 2018 and March 2021 were included. According to mRS scores after three months, a total of 140 patients were divided into the good prognosis group(n=115) (mRS score <2) and the poor prognosis group(n=25) (mRS score ≥2).

### Ethical Approval

The study was approved by the Institutional Ethics Committee of Baoding No.1 Central Hospital on April 15, 2022(No.:2022021), and written informed consent was obtained from all participants.

### Inclusion criteria:

- Patients aged >18 years;
- Patients with an mRS score ≤1 before the onset of stroke;
- Patients without a history of hemorrhagic stroke, and clinically considered to have an ischemic stroke and with a baseline NIHSS score ≤5 when visiting our hospital;
- Patients who received intravenous thrombolysis with alteplase in our hospital, had intracranial hemorrhage excluded by cranial CT before thrombolysis, did not have imaging manifestations of early massive cerebral infarction, had cranial CT reexamined 24 hour after thrombolysis, or had cranial CT examination when the condition worsened;
- Patients with complete medical history, imaging and laboratory data;
- Patients who had received outpatient follow-up or telephone follow-up three months after the onset of the disease with complete follow-up data.

### Exclusion criteria:

- Patients meeting the absolute contraindications to thrombolysis in the Chinese Guidelines for the Diagnosis and Treatment of Acute Ischemic Stroke 2018;
- Patients undergoing endovascular bridging therapy or decompressive craniectomy/ventricular drainage;
- Patients with severe cardiac, hepatic, or renal insufficiency;
- Women during pregnancy;
- Patients with dementia, mental disorder or inability to cooperate with treatment;
- Patients with a definite history of infectious diseases in the last two weeks, or with autoimmune diseases, blood system diseases (Iron deficiency anemia, acute myeloid leukemia, abnormal coagulation function, etc.), or who have taken steroid hormones, immunosuppressants, chemotherapy drugs and other drugs that may affect platelet index;
- Patients with severe hypertension (Systolic blood pressure in excess of 180mmHg, or diastolic blood pressure in excess of 110mmHg);
- Patients with severe end-stage diseases such as tumors;
- Patients with severe trauma in the last two weeks;
- Patients with pseudo-stroke;

### Treatment methods

Atelipases (Actilyse) 20 mg/injection (Boehringer-Ingelheim, Germany, Approval No.: S20110051) or 50mg/injection (Boehringer-Ingelheim, Germany, Approval No.: S20110052) was administered. Atelipases were administered at a dose of 0.9 g/kg (maximum dose 90 mg), dissolved to a concentration of one mg/mL. Ten percent was injected within one minute, and 90% was intravenously dripped within one hour. The use of antiplatelet and anticoagulant drugs, including aspirin, clopidogrel, tegrello, tirofiban, warfarin and low molecular weight heparin, was strictly prohibited within 24 hour after intravenous thrombolysis. Head CT was routinely reexamined at 24 h, and after excluding cerebral hemorrhage, antiplatelet therapy was given according to “Guidelines for Diagnosis and Treatment of Acute Ischemic Stroke in China 2018”, and statins and drugs for improving microcirculation were routinely given.

### Data collection

Medical records were reviewed to collect patients’ basic information, vascular risk factors, medication history, door-to-needle time (DNT), onset-to-door time (OTT), baseline blood pressure, baseline NIHSS score, 24 h NIHSS score after thrombolysis, infarction area, stenosis or occlusion of aorta, etiological classification of cerebral infarction and laboratory data.

### Prognosis assessment

The primary prognostic indexes of the study were expressed by mRS scores three months after onset, and were assessed by outpatient follow-up or telephone follow-up by trained professionals. An mRS score <two was considered a good prognosis, and a score of two as a poor prognosis). Secondary prognostic indexes included early neurological deterioration (END), hemorrhagic transformation (HT) and symptomatic intracranial hemorrhage. Among these, END was defined as an increase in NIHSS score ≥four points from baseline NIHSS score at 24 hour after intravenous thrombolysis^9^; HT was defined as no hemorrhage in the skull CT before intravenous thrombolysis, but any visible intracranial hemorrhage is found in the subsequent skull CT examination^10^; Symptomatic intracranial hemorrhage (sICH) was defined as intracranial hemorrhage resulting in worsening clinical symptoms, an increase in NIHSS score of ≥four points, or resulting in death.^11^

### Statistical analysis

All data in this study were analyzed using SPSS 23.0 statistical software. The measurement data of single factor analysis which is in accordance with normal distribution was expressed by mean standard deviation (*χ̅*±S), and independent samples t-test was used for comparison between groups. The measurement data inconsistent with the normal distribution were represented by the median (quartile) [M(P_25_, P_75_)], and Mann-Whitney test was used for comparison between groups; The data were expressed by frequency (percentage), and the comparison between groups was made by s^2^ test or Fisher exact probability method. Factors with P< 0.1 for single-factor analysis were included in multivariate analysis, and Logistic regression analysis was adopted, with P<0.05 was considered statistically significant for multivariate analysis. ROC curves were selected for the predictive analysis.

## RESULTS

The comparison of clinical, imaging and laboratory data between the two groups. Table-I History of diabetes, aortic stenosis, baseline NIHSS score, 24 hour NIHSS score, and MPV were further included for logistic regression analysis based on the results of univariate analysis. Increased 24 hour NIHSS score and increased baseline MPV were independent risk factors for poor prognosis as shown in Table-II.

**Table-I:** Comparison of clinical, imaging and laboratory data between patients with good three months prognosis and those with poor 3-month prognosis.

		Three months prognosis		P value

		Good prognosis (n=115)	Poor prognosis (n=25)	
Gender (male)		71 (61.7%)	15 (60.0%)	0.871^a^
Age (years old)		61.5±11.5	61.6±13.6	0.962^b^
Weight (kg)		71.7±11.7	70.1±13.4	0.554^b^
BMI (kg/m^2^)		25.7±3.6	25.3±3.8	0.661^b^
Family history (n%)	Hypertension	10 (8.7%)	1 (4.0%)	0.689^c^
	Diabetes	2 (1.7%)	1 (4.0%)	0.448^c^
	Cardiovascular and cerebrovascular diseases	11 (9.6%)	3 (12.0%)	0.716^c^
History of smoking (n%)		48 (41.7%)	10 (40.0%)	0.873^a^
History of alcohol consumption (n%)		38 (33.0%)	7 (28.0%)	0.625^a^
Medical history (n%)	Hypertension	76 (66.1%)	16 (64.0%)	0.842^a^
	Diabetes	21 (18.3%)	11 (44.0%)	0.005^a^
	Coronary heart disease	29 (25.2%)	6 (24.0%)	0.899^a^
	Ischemic stroke	27 (23.5%)	8 (32.0%)	0.372^a^
Atrial fibrillation (n%)		10 (8.7%)	3 (12.0%)	0.703^c^
Medication history (n%)	Antiplatelet drug	20 (17.5%)	5 (20.0%)	0.777^c^
	Anticoagulant	0 (0%)	0 (0%)	-
	Hypotensor	57 (49.6%)	14 (56.0%)	0.560^a^
	Hypoglycemic drug	18 (15.7%)	7 (28.0%)	0.156^c^
DNT (min)		45.0 (34.0,64.0)	43.0 (34.0,47.0)	0.143^d^
OTT (min)		144.2±57.4	136.4±49.7	0.530^b^
Baseline blood pressure	Systolic blood pressure (mmHg)	151.6±19.7	153.0±23.7	0.753^b^
	Diastolic blood pressure (mmHg)	88.0 (80.0,95.0)	88.0 (81.0,98.0)	0.723^d^
Baseline NIHSS score		2.0 (1.0,4.0)	3.0 (2.0,5.0)	0.002^d^
24hNIHSS score after thrombolysis		1 (0.0,2.0)	7.0 (4.5,9.5)	0.000^d^
Massive cerebral infarction (n%)		2 (1.7%)	0 (0%)	1.000^c^
Arterial stenosis (≥50%) or occlusion (n%)		19 (16.5%)	8 (32.0%)	0.094^c^
TOAST typing (n%)				0.301^c^
Large-artery atherosclerosis		20 (17.4%)	7 (28.0%)	-
Small-artery occlusion		87 (75.7%)	15 (60.0%)	-
Cardiogenic		5 (4.3%)	2 (8.0%)	-
Etiology unknown		3 (2.6%)	1 (4.0%)	-
Hcy (μmol/L)		11.92 (9.64,17.31)	12.41 (10.05,16.14)	0.870^d^
Baseline blood glucose (mmol/L)		7.01 (5.83,8.48)	7.64 (6.27,12.67)	0.130^d^
HbAlc (%)		5.80 (5.50,6.30)	5.90 (5.60,8.60)	0.156^d^
ALB (g/L)		43.89±3.82	42.54±3.39	0.106^b^
ALT (U/L)		23.20 (19.20,33.50)	23.10 (19.20,30.20)	0.695^d^
AST (U/L)		23.30 (20.10,27.20)	24.40 (17.45,26.50)	0.759^d^
CREA (μmol/L)		65.60 (57.00,78.10)	59.50 (48.70,70.70)	0.170^d^
UA (μmol/L)		342.66±83.69	334.87±107.09	0.690^b^
CHO (mmol/L)		4.66±0.97	4.94±1.03	0.193^b^
LDL-C (mmol/L)		2.75 (2.10,3.28)	2.81 (2.56,3.46)	0.172^d^
HDL-C (mmol/L)		1.10±0.25	1.12±0.25	0.736^b^
TG (mmol/L)		1.33 (0.91,2.00)	1.22 (0.87,1.92)	0.621^d^
PT (s)		10.30 (10.00,10.70)	10.30 (10.05,10.60)	0.614^d^
APTT (s)		25.83±2.28	25.43±2.15	0.436^b^
Fg (g/L)		2.77 (2.33,3.20)	2.64 (2.31,2.94)	0.507^d^
D-D (mg/L)		0.21 (0.15,0.36)	0.22 (0.16,0.50)	0.488^d^
HGB (g/L)		145.56±14.54	145.68±17.76	0.971^b^
PC (×10^9^)		223.00 (190.00,258.00)	232.00 (179.00,273.00)	0.769^d^
MPV (fL)		9.77±0.70	10.16±0.71	0.013^b^
PDW (fL)		10.70 (10.10,11.70)	11.50 (10.00,12.70)	0.144^d^
WBC count (×10^9^)		7.42 (5.83,8.72)	7.40 (5.88,8.52)	0.862^d^
Neutrophil count (×10^9^)		4.21 (3.25,5.39)	4.47 (3.40,5.41)	0.771^d^
Lymphocyte count (×10^9^)		2.15 (1.67,2.84)	1.75 (1.32,2.59)	0.176^d^

***Note:*** “a” means *χ*^2^ test data, “b” means t test data, “c” means Fisher exact test data, “d” means Mann-Whitney test, “-” means no data.

**Table-II:** Multivariate Logistic regression analysis of poor prognosis three months after onset.

Variables	B	SE	Wald^2^	P value	OR	95%CI
Baseline NIHSS score	-0.499	0.414	1.453	0.228	0.607	0.269-1.367
24hNIHSS score after thrombolysis	1.251	0.334	13.997	0.000	3.494	1.814-6.729
History of diabetes	-0.830	0.785	1.116	0.291	0.436	0.094-2.033
Arterial stenosis (≥50%) or occlusion (n%)	-0.407	0.919	0.196	0.658	0.666	0.110-4.031
MPV	1.254	0.586	4.581	0.032	3.503	1.111-11.040
Constant	-15.917	6.096	6.818	0.009	0.000	-

There were 11 patients with early neurological deterioration, three patients with hemorrhagic transformation, one patient with symptomatic intracranial hemorrhage, and no patient died within three months. The comparison results of clinical, imaging and laboratory data between patients with END and those without END are shown in Table-III. Logistic regression analysis was performed by including MPV and NEUT, which were included in the univariate analysis at P<0.1, and showed that the increase of MPV also increased the risk of END. The results of multivariate regression analysis are shown in Table-IV.

**Table-III:** Univariate analysis of END after intravenous thrombolysis in patients with acute mild cerebral infarction.

		END		P value

		Yes (n=11)	No (n=129)	
Gender (Male)		7 (63.6%)	79 (61.2%)	1.000^a^
Age (Years old)		55.4±13.0	62.1±11.7	0.072^b^
Weight (kg)		74.0±15.3	71.2±11.7	0.453^b^
BMI (kg/m^2^)		26.2±4.4	25.6±3.5	0.548^b^
Family history (n%)	Hypertension	0 (0.0%)	11 (8.5%)	0.601^a^
	Diabetes	0 (0.0%)	3 (2.3%)	1.000^a^
	Cardiovascular and cerebrovascular diseases	1 (9.1%)	13 (10.1%)	1.000^a^
History of smoking (n%)		4 (36.4%)	54 (41.9%)	1.000^a^
History of alcohol consumption (n%)		3 (27.3%)	42 (32.6%)	1.000^a^
Medical history (n%)	Hypertension	8 (72.7%)	84 (65.1%)	0.749^a^
	Diabetes	4 (36.4%)	28 (21.7%)	0.274^a^
	Coronary heart disease	2 (18.2%)	33 (25.6%)	0.731^a^
	Ischemic stroke	4 (36.4%)	31 (24.0%)	0.467^a^
Atrial fibrillation (n%)		1 (9.1%)	12 (9.3%)	1.000^a^
Medication history (n%)	Antiplatelet drug	1 (9.1%)	24 (18.8%)	0.689^a^
	Anticoagulant	0 (0%)	0 (0%)	-
	hypotensor	7 (63.6%)	64 (49.6%)	0.372^c^
	Hypoglycemic drug	3 (27.3%)	22 (17.1%)	0.414^a^
DNT (min)		37.0 (30.0,46.0)	45.0 (34.0,63.0)	0.207^d^
OTT (min)		126.5±54.2	144.1±56.1	0.319^b^
Baseline blood pressure	Systolic blood pressure (mmHg)	154.8±25.1	151.6±20.0	0.619^b^
	Diastolic blood pressure (mmHg)	90.0 (79.0,110.0)	88.0 (80.0,95.0)	0.473^d^
Baseline NIHSS score		3.0 (2.0,5.0)	2.0 (1.0,4.0)	0.112^d^
Massive cerebral infarction (n%)		0 (0%)	2 (1.6%)	1.000^a^
Arterial stenosis (≥50%) or occlusion (n%)		4 (36.4%)	23 (17.8%)	0.223^a^
TOAST typing (n%)				0.559^a^
Large-artery atherosclerosis		3 (27.3%)	24 (18.6%)	-
Small-artery occlusion		7 (63.6%)	95 (73.6%)	-
Cardiogenic		1 (9.1%)	6 (4.7%)	-
Etiology unknown		0 (0.0%)	4 (3.1%)	-
Hcy (μmol/L)		12.41 (10.65,15.38)	11.92 (9.71,17.11)	0.615^d^
Baseline blood glucose (mmol/L)		6.40 (6.19,15.24)	7.03 (5.87,8.61)	0.727^d^
HbAlc (%)		5.70 (5.40,9.30)	5.80 (5.55,6.40)	0.831^d^
ALB (g/L)		42.74±3.58	43.73±3.79	0.405^b^
ALT (U/L)		24.00 (21.20,34.50)	23.10 (19.10,32.70)	0.464^d^
AST (U/L)		24.40 (17.80,29.80)	23.30 (19.45,26.80)	0.690^d^
CREA (μmol/L)		58.00 (52.80,68.60)	64.70 (55.35,78.80)	0.312^d^
UA (μmol/L)		349.18±114.75	340.59±85.82	0.757^b^
CHO (mmol/L)		4.78±1.20	4.70±0.97	0.791^b^
LDL-C (mmol/L)		2.64 (2.22,3.37)	2.78 (2.19,3.28)	0.911^d^
HDL-C (mmol/L)		1.15±0.28	1.10±0.25	0.464^b^
TG (mmol/L)		1.14 (0.73,1.83)	1.37 (0.92,2.03)	0.149^d^
PT (s)		10.50 (10.20,10.90)	10.30 (10.00,10.70)	0.223^d^
APTT (s)		25.48±1.74	25.78±2.30	0.676^b^
Fg (g/L)		2.68 (2.26,3.25)	2.77 (2.34,3.17)	0.843^d^
D-D (mg/L)		0.20 (0.12,0.32)	0.22 (0.15,0.37)	0.529^d^
HGB (g/L)		152.73±13.58	144.97±15.11	0.102^b^
PC (×10^9^)		233.00 (181.00,289.00)	223.00 (187.00,258.00)	0.561^d^
MPV (fL)		10.25±0.86	9.81±0.70	0.052^b^
PDW (fL)		11.30 (10.20,13.50)	10.70 (10.10,11.75)	0.282^d^
WBC count (×10^9^)		7.55 (6.69,8.96)	7.41 (5.80,8.51)	0.227^d^
Neutrophil count (×10^9^)		5.06 (4.44,6.03)	4.18 (3.24,5.36)	0.068^d^
Lymphocyte count (×10^9^)		1.65 (1.34,2.82)	2.11 (1.65,2.83)	0.500^d^

***Note:*** “a” means *χ*^2^ test data, “b” means t test data, “c” means Fisher exact test data, “d” means Mann-Whitney test, “-” means no data.

**Table-IV:** Multivariate Logistic regression analysis of END after intravenous thrombolysis in patients with acute mild cerebral infarction.

Variables	B	SE	Wald^2^	P value	OR	95%CI
Age	-0.056	0.029	3.811	0.051	0.945	0.894-1.000
Neutrophil count	0.173	0.153	1.279	0.258	1.189	0.881-1.605
MPV	1.129	0.492	5.274	0.022	3.094	1.180-8.112
Constant	-11.342	5.229	4.704	0.030	0.000	-

**Fig.1:**
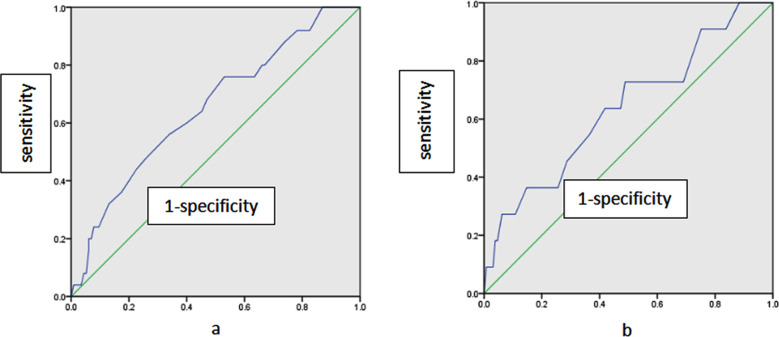
ROC curves for the ROC curves for the 3-month adverse prognosis and END in patients with acute mild cerebral infarction undergoing intravenous thrombolysis. (a) ROC curve of 3-month poor prognosis of patients with acute mild cerebral infarction undergoing intravenous thrombolysis; (b) ROC curve of END of patients with acute mild cerebral infarction undergoing intravenous thrombolysis.

ROC curves were used to analyze the predictive value of MPV for three-month poor prognosis and END. Fig.1 shows two ROC curves. Table-V shows an optimal cutoff value of 9.65 fl for MPV in predicting three-month poor prognosis, and an optimal cutoff value of 9.75 fl for MPV in predicting END.

**Table-V:** ROC curve analysis of MPV on three months adverse prognosis and END in patients with acute mild cerebral infarction undergoing intravenous thrombolysis.

Outcomes	AUC	95%CI	Cutoff value (fl)	Sensitivity (%)	Specificity (%)
Unfavorable outcome after 3 months	0.653	0.535-0.770	9.65	76.0	47.0
END	0.637	0.460-0.814	9.75	72.7	51.2

## DISCUSSION

Mean Platelet Volume(MPV) is commonly used and convenient indicator to reflect platelet size and platelet activation, and platelet activation is recognized to play a vital role in the pathological process of ischemic cerebrovascular disease. In this study, the relationship between MPV and prognosis of intravenous thrombolysis in patients with acute mild ischemic stroke was investigated. It was shown in this study that MPV is an independent risk factor for poor prognosis and early neurological deterioration in patients with acute mild cerebral infarction three months after thrombolysis. It has been found in many studies that MPV has a close bearing on the occurrence and severity of ischemic stroke.^12^,^13^ Pikija et al.^14^ found that the increase of MPV can indicate a larger infarct volume and a worse prognosis on skull CT. Du et al.^15^ found that MPV is an independent risk factor for ischemic stroke and hemorrhagic stroke, and is independently related to the poor prognosis of ischemic stroke and hemorrhagic stroke. In recent years, there have been many research reports on MPV and prognosis of patients with acute cerebral infarction undergoing intravenous thrombolysis. Dourado et al.^6^ revealed that MPV is independently related to poor functional prognosis at discharge, although MPV was not an independent risk factor for poor functional prognosis at 90 days, MPV of patients with poor functional prognosis at 90 days also tended to increase. Yao et al.^16^ found that MPV was an independent risk factor for poor prognosis in three months. However, there is no limit to the NIHSS score in these studies, including both patients with mild stroke and patients with high NIHSS score. In addition, recent studies have shown that quite a number of patients with acute mild cerebral infarction have deteriorated neurological function and poor prognosis.^17^

There are currently few international reports on the relationship between MPV and hemorrhagic transformation of cerebral infarction. In this study, there were three cases (2.1%) of hemorrhagic transformation, and zero mortality within three months. Logistic regression analysis could not be performed due to insufficient number of cases, but these data also suggest that the risk of bleeding transformation and the mortality of patients with mild ischemic stroke after intravenous thrombolysis may not be high. Wang et al.^18^ studied 783 patients with ischemic stroke and found that MPV is an independent risk factor for hemorrhagic transformation, with the increase of MPV, the risk of hemorrhagic transformation increases nonlinearly. In analyzing the relationship between MPV and prognosis, Dourado et al^6^ explored the relationship between platelet indexes such as MPV and PDW and hemorrhagic transformation. They found that only PDW was related to severe hemorrhagic transformation, but this relationship was not statistically significant after the interferenormation after intravenous thrombolysis.

According to the results of our study, MPV can be used as an important index to predict the prognosis of acute mild ischemic stroke after intravenous thrombolysis, and it is convenient and easy to measure, which has important value for predicting the prognosis and guiding clinical treatment.

### Limitations

It includes a small sample size and a retrospective study. Secondly, some potential factors that affect prognosis may not be included in the study. Finally, only the association of MPV with poor prognosis at three months was analyzed, and perhaps longer follow-up could be performed.

## CONCLUSIONS

Baseline MPV is an independent risk factor for early neurological deterioration and poor prognosis for three months in patients with intravenous thrombolysis for acute mild cerebral infarction, and it is of great significance for prognosis prediction and clinical decision-making.

### Authors’ Contributions:

**YW and XW** carried out the studies, participated in collecting data, and drafted the manuscript, and are responsible and accountable for the accuracy and integrity of the work.

**WD** performed the statistical analysis and participated in its design.

**CY** participated in acquisition, analysis, or interpretation of data and draft the manuscript. All authors read and approved the final manuscript.
